# Excretion of the Polymyxin Derivative NAB739 in Murine Urine

**DOI:** 10.3390/antibiotics9040143

**Published:** 2020-03-27

**Authors:** Martti Vaara, Timo Vaara, Janis Kuka, Eduards Sevostjanovs, Solveiga Grinberga, Maija Dambrova, Edgars Liepinsh

**Affiliations:** 1 Northern Antibiotics Ltd., FI-02150 Espoo, Finland; martti.vaara@northernantibiotics.com (M.V.); timo.vaara@northernantibiotics.com (T.V.); 2 Department of Bacteriology and Immunology, Helsinki University Medical School, FI-00014 Helsinki, Finland; 3 Laboratory of Pharmaceutical Pharmacology, Latvian Institute of Organic Synthesis, Aizkraukles Str 21, LV1006 Riga, Latvia; janis.kuka@farm.osi.lv (J.K.); eduards@osi.lv (E.S.); solveiga@osi.lv (S.G.); maija.dambrova@farm.osi.lv (M.D.)

**Keywords:** NAB739, polymyxin B, mouse pyelonephritis, extremely multiresistant strains of Enterobacteriaceae

## Abstract

Extremely multiresistant strains of Enterobacteriaceae are emerging and spreading at a worrisome pace. Polymyxins are used as the last-resort therapy against such strains, in spite of their nephrotoxicity. We have previously shown that novel polymyxin derivatives NAB739 and NAB815 are less nephrotoxic in cynomolgus monkeys than polymyxin B and are therapeutic in murine *Escherichia coli* pyelonephritis at doses only one-tenth of that needed for polymyxin B. Here we evaluated whether the increased efficacy is due to increased excretion of NAB739 in urine. Mice were treated with NAB739 and polymyxin B four times subcutaneously at doses of 0.25, 0.5, 1, 2, and 4 mg/kg. In plasma, a clear dose–response relationship was observed. The linearity of C_max_ with the dose was 0.9987 for NAB739 and 0.975 for polymyxin B. After administration of NAB739 at a dose of 0.25 mg/kg, its plasma concentrations at all tested time points were above 0.5 µg/mL while after administration at a dose of 0.5 mg/kg its plasma concentrations exceeded 1 µg/mL. The C_max_ of NAB739 in plasma was up to 1.5-times higher after single (first) administration and up to two-times higher after the last administration when compared to polymyxin B. Polymyxin B was not detected in urine samples even when administered at 4 mg/kg. In contrast, the concentration of NAB739 in urine after single administration at a dose of 0.25 mg/kg was above 1 µg/mL and after administration of 0.5 mg/kg its average urine concentration exceeded 2 µg/mL. At the NAB739 dose of 4 mg/kg, the urinary concentrations were higher than 35 µg/mL. These differences explain our previous finding that NAB739 is much more efficacious than polymyxin B in the therapy of murine *E. coli* pyelonephritis.

## 1. Introduction

Extremely multiresistant strains of Enterobacteriaceae, such as those of *Escherichia coli* and *Klebsiella pneumoniae*, are emerging and spreading at a worrisome pace. In the last few years also the species *Proteus mirabilis* has emerged as a frequent and important cause of the onset of urinary tract infections (UTIs) [1,2]. Two polymyxin class antibiotics, colistin and polymyxin B (PMB), are currently used as the last-resort therapy against multidrug resistant strains, in spite of their nephrotoxicity. Unfortunately, polymyxins are highly nephrotoxic agents which frequently induce acute kidney injury at conventional doses and may require discontinuation of the therapy [3]. Quite recently, international consensus guidelines have been published on the optimal use of polymyxins [3]. Numerous efforts have been made to develop derivatives of polymyxins that are less nephrotoxic than those now in clinical use. Another approach is designing of new polymyxin derivatives that are more effective than the old ones and therefore could be used at lower, better tolerated doses. An excellent and comprehensive review on those attempts has recently been published [4].

**Figure 1:**
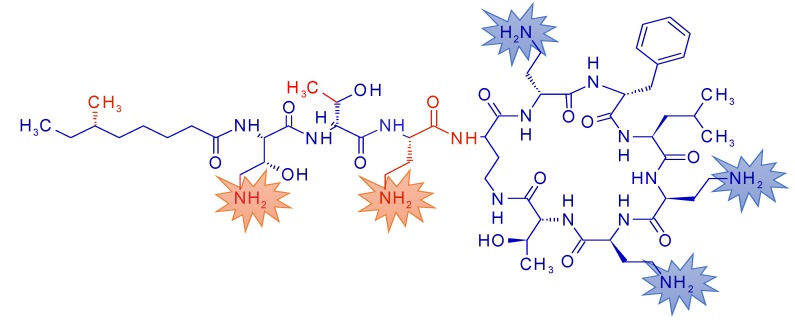
The structure of polymyxin B2 and NAB739. Polymyxin B2-specific functional groups are indicated with red font. Stars outline charged –NH_2_ groups.

Polymyxins are cyclic lipopeptides that have a cyclic heptapeptide part and a linear tripeptide part, linked to a fatty acyl moiety. Colistin and PMB are highly charged because of five free positively charged amino groups, three of them in the cyclic part and two in the linear part of the molecule. Depending on localization of cationic functional groups, both efficacy and nephrotoxicity of polymyxins is due to the highly cationic nature of the molecule [5]. We have synthesized polymyxin derivatives that carry only three positive charges (Figure 1) [6,7]. NAB739 does not carry any positive charge in the linear peptide part. NAB815 carries only one positive charge in the linear part and two positive charges in the cyclic part. In cynomolgus monkeys both novel polymyxins are less nephrotoxic than PMB. In addition, unlike PMB, both NAB compounds are excreted in the cynomolgus urine at a very significant extent [6,7]. Additionally, in patients, only less than 1% of PMB dose is excreted in urine which significantly limits their use for the treatment of urinary tract infections [8].

In a murine model of *E. coli* pyelonephritis, both NAB739 and NAB815 reduced the bacterial burden in the kidney, urine, and bladder at doses approximately 10-fold lower than those of PMB [9]. In kidneys, the half maximal effective dose (ED_50_) was 0.24 mg/kg for NAB739 and 2.1 mg/kg for PMB. We hypothesized that the substantially increased efficacy might be related to increased urinary levels of new polymyxins. Therefore, we studied the comparative urinary levels of NAB739 and PMB in mice using various doses to show that sufficient-for-efficacy amounts of NAB739 are excreted in the urine whereas PMB is not present in the murine urine even after the treatment at the highest dose of 4 mg/kg.

## 2. Materials and Methods

### 2.1. Methods

NAB739 sulfate was custom-made by Bachem AG (Bubendorf, Switzerland). Its purity, as estimated by HPLC, was 97.3%. Polymyxin B (PMB) sulfate was from Sigma-Aldrich (St. Louis, MO, USA; product number PO0972). Sterile sodium chloride 0.9% solution was obtained from Fresenius Kabi (Bad Homburg, Germany). Microvette for capillary blood collection was from Sarstedt (Nümbrecht, Germany). Hydrophobic sand LabSand for urine sample collection was from Datesand Group (Manchester, UK).

### 2.2. Animals

NMRI female mice (10–12 weeks old, 29–32 g, *n* = 64) were obtained from ENVIGO (The Netherlands) and housed prior to treatment under standard conditions (acclimatization period of 1 week, 21–23 °C, 12-h light/dark cycle, relative humidity 45%–65%) with unlimited access to food (R70 diet from Lantmännen) and water. The experimental procedures were performed in accordance with the guidelines of the European Community and local laws and policies (Directive 2010/63/EU), and all of the procedures were approved by Food and Veterinary Service, Riga, Latvia. Studies involving animals are reported in accordance with the ARRIVE guidelines [10,11]. Animals were weighed on the day of the treatment (before treatment) to calculate the required amount of compound for the corresponding dose. For PK and excretion studies the mice were dosed subcutaneously (SC) via a bolus injection (injection volume 10 mL/kg). Four consecutive doses were administrated twice a day at 9:00 AM and 15:00 PM over the course of two days.

Blood samples were collected in tubes containing heparin. Blood was sampled from tail vein 15, 30, and 45 min after the first and last administrations of the compounds. Tubes were centrifuged at +4 °C 10,000× *g* for 3 min. Plasma samples were collected and stored at −20 °C until analysis. 

For PK study, before the administration of the compound, mice voiding was stimulated. Then compound was administrated SC. Mice were put in separate cages with LabSand (USA) and urine was collected during the next 3 h. At the end of each 1-h period voiding was stimulated by gentle massage of the belly. Urine samples from each mouse were pooled in one tube. Urine volume was determined by weighing of the pooled urine sample.

### 2.3. Plasma and Urine Sample Analysis

Plasma and urine sample analyses were performed using the quantitative UPLC/MS/MS method. Calibration standards and quality control samples for plasma analysis were prepared by spiking blank mouse plasma (CD-1, anticoagulant Na Heparin, Innovative Research, USA) with a stock solution of analyte to achieve a nominal concentration ranging from 0.01 to 50 μg/mL for NAB739 and PMB. Calibration standards and quality control samples for urine analysis were prepared by spiking blank mouse urine (mouse CD-1 urine, Sera Laboratories International Ltd., UK) with a stock solution of analytes to achieve a nominal concentration from 0.04 to 30 μg/mL for NAB739 and PMB.

A sample volume of 20 μL (calibration standards, quality controls or analytical samples in plasma or urine) was transferred into the plastic tube and mixed with 100 μL of 1% formic acid solution in acetonitrile/methanol (3:1, v/v) mixture to precipitate proteins. The tubes were vortex- mixed and centrifuged at 10,000 rpm for 10 min. Then, 100 μL of supernatant was diluted with 400 μL of water and subjected to UPLC/MS analysis as follows:

UPLC system Acquity H-class (Waters) connected with triple quadrupole mass spectrometer Xevo TQ-S (Waters). UPLC conditions: Column—Acquity BEH C18 (2.1 × 50 mm, 1.7 µm); Mobile phase—A: 0.1% Formic acid aqueous solution, B: Acetonitrile; Gradient: Initial—90%A, 2.5 min—5% A, 4.0 min—5% A, 4.5 min—90% A, 6 min—90% A; Flow—0.3 mL/min; Column temperature—40 °C; Injection volume—1 μL (NAB739) or 5 μL (PMB). MS conditions: Ionization—ESI positive mode; Capillary voltage—1.0 kV; ESI source temperature—120 °C; Desolvation gas (N2) flow—800 L/h; Desolvation temperature—600 °C.

## 3. Results

Quantitative UPLC/MS/MS method is suitable for the determination of PMB and NAB739 in mouse plasma in the concentration range from 0.02 to 16 μg/mL and in mouse urine in the concentration range from 0.58 to 16 μg/mL. Performance of polymyxin analysis method and MRM parameters for NAB739 and Polymyxin B are described in Table 1 and Table 2.

### 3.1. Pharmacokinetic Profiles of NAB739 and PMB

The plasma concentrations of NAB739 and PMB were determined following single-dose and four-times-repeated administration of compounds at doses of 0.25, 0.5, 1, 2, and 4 mg/kg. During the administration of compounds, no adverse effects were observed at any of the tested doses.

**Figure 2:**
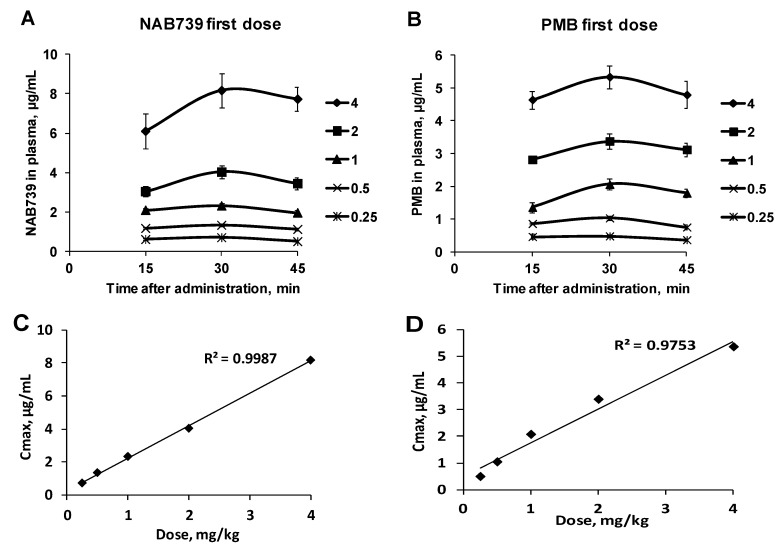
Concentrations of compounds in NMRI mice plasma after the first SC administration of NAB739 (**A**) and PMB (**B**) at doses of 0.25–4mg/kg. Dose and C_max_ relationship of NAB739 (**C**) and PMB (**D**) at doses of 0.25–4 mg/kg. Data expressed as average ± SEM from four mice.

After SC administration of NAB739 and PMB clear dose–response relationship for doses from 0.25 to 4 mg/kg was observed (Figure 2). C_max_ values for both compounds and at all doses were observed 30 min after the administration. The linearity of C_max_ and a dose–response relationship was slightly better for NAB739 than for PMB, correlation coefficients being 0.9987 and 0.975, respectively (Figure 2C,D). After administration of NAB739 at a dose of 0.25 mg/kg its plasma concentrations at all time points were above 0.5 µg/mL while after administration at a dose of 0.5 mg/kg plasma concentrations exceeded 1 µg/mL.

**Figure 3:**
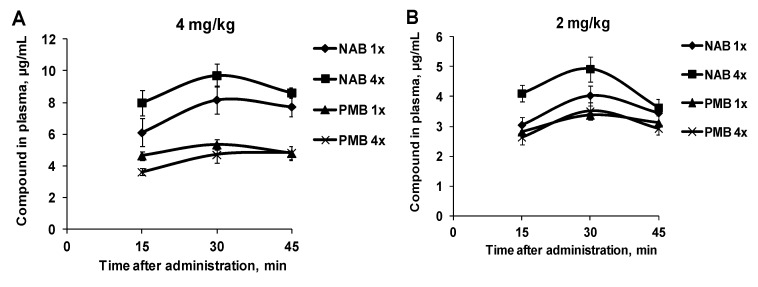

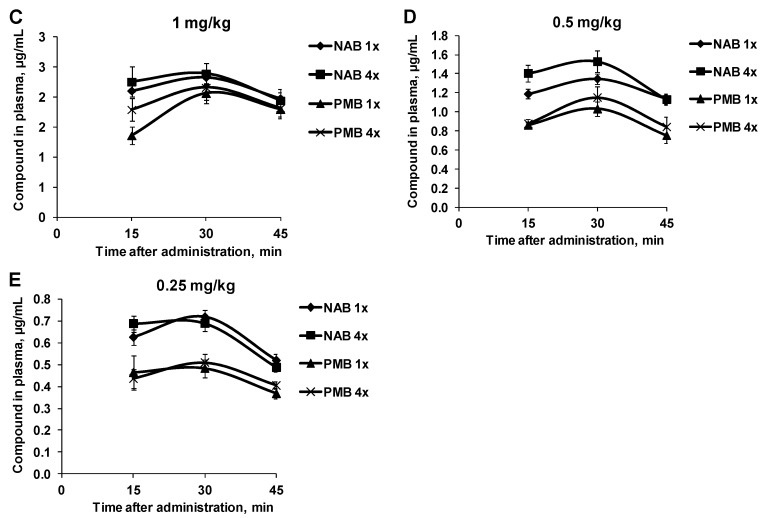
Comparison of PK profiles in NMRI mice plasma after the first and the last SC administration of NAB739 and PMB at doses of 4 (**A**), 2 (**B**), 1 (**C**), 0.5 (**D**), 0.25 (**E**) mg/kg. Data expressed as average ± SEM from four mice.

At all tested doses after both single and repeated dosing NAB739 reached higher plasma concentrations than PMB (Figure 3) indicating a better bioavailability of NAB739. C_max_ of NAB739 was up to 1.5-times higher after single (first) administration and up to two-times higher after the last (fourth) administration when compared to PMB (Figure 3). Plasma concentrations of PMB were similar after the first and the last administration of the compound, while concentrations of NAB739 were up to 30% higher after the last administration of the compound at doses of 2 and 4 mg/kg (Figure 3). 

### 3.2. Urinary Elimination of NAB739 and PMB

**Figure 4:**
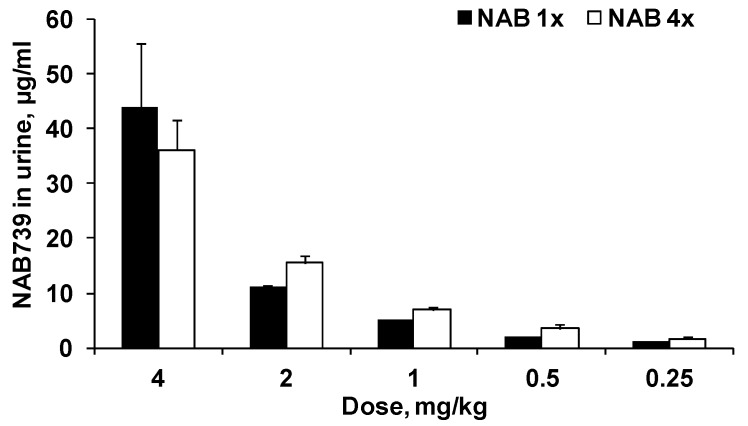
Concentrations of NAB739 in NMRI mice urine after the first and the last SC administration at doses of 0.25–4 mg/kg. Data expressed as average ± SEM from four NMRI mice.

PMB was not detected in urine samples even when the compound was administered at a dose of 4 mg/kg (data not shown). NAB739 was found in all pooled (0–3 h) urine samples (Figure 4). After administration of NAB739 at doses of 0.25–2 mg/kg urine concentrations of the compound on average were 27% (5.1%–49%) higher after the last dose compared to concentration after the first dose (Figure 4). The concentration of NAB739 in urine after single administration at a dose of 0.25 mg/kg was above 1 µg/mL while after administration of 0.5 mg/kg average urine concentration exceeded 2 µg/mL.

**Figure 5:**
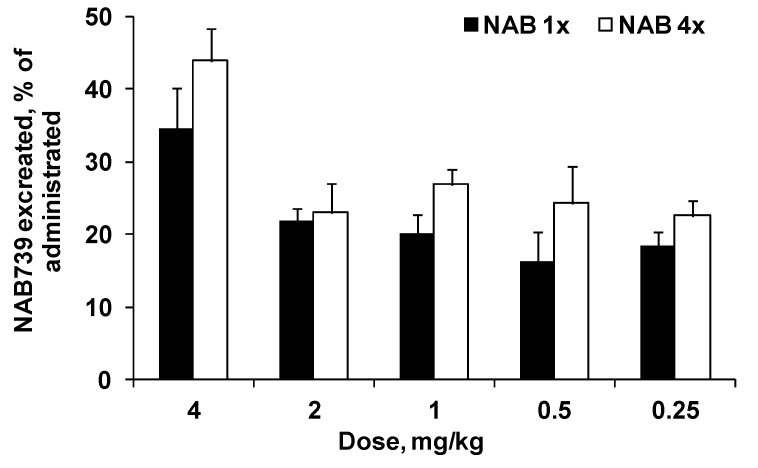
Relative amounts of compound eliminated by urine after the first and the last SC administration of NAB739 at doses of 0.25–4 mg/kg. Relative amounts expressed as a percentage of the amount of compound administrated in mice at particular dose. Data expressed as average ±SEM from four NMRI mice.

As shown in Figure 5, the relative elimination of NAB739 by urine was similar for all doses. On average, about 20% of the compound was eliminated by urine during 3 h after the first administration of NAB739 at doses of 0.25–2 mg/kg. At a dose of 4 mg/kg about 35% of administered compound was eliminated by urine. After administration of the last dose, relative elimination of NAB739 was on average 26% (5.1%–49%) higher than after the administration of the first dose of the compound.

## 4. Discussion

We showed here that even though subcutaneous administration of NAB739 and PMB yielded similar plasma concentrations in mice, only NAB739 was eliminated into urine at reasonable quantities. After the fourth dose of PMB at a dose of 4 mg/kg, plasma level was above 6 µg/mL, but compound was not found in urine (detection limit, 0.6 µg/mL). In contrast, even at the doses of 0.25 and 0.5 mg/kg, the concentration of NAB739 in urine was above 1 and 2 µg/mL, respectively. Higher doses yielded very high NAB739 concentrations in urine, thus after a dose of 4 mg/kg, the urinary concentrations of NAB739 were above 35 µg/mL. These differences explain our previous finding that NAB739 is much more efficacious than PMB for the treatment of murine *E. coli* pyelonephritis. 

The molecular mechanism behind our finding that NAB739 and NAB815, but not PMB, are excreted in urine, is not known. We have previously shown that compared to PMB, NAB polymyxin derivatives with three positive charges are much less bound to the brush border membrane of the kidney proximal tubule (PT) cells of rats [12]. This is likely since, in contrast to PMB with five positive charges, the NAB derivatives have only three positive charges and could, therefore, be less prone to interact with the negatively charged lipids mostly found in the cytosolic leaflet of the plasma membrane [13]. It may be possible that polymyxin B is reabsorbed (taken up) by the PT cells at a rate much higher than that for the less charged derivatives.

Pyelonephritis is a complicated kidney-affecting urinary tract infection (cUTI) that can lead to septicemic blood stream infection, if not properly treated. Currently, PMB and colistin are used in humans. Colistin is administered intravenously as its bacteriologically inactive prodrug colistin methanesulfonate (CMS), also known as colistimethate [3]. Most of colistin is excreted into urine as its prodrug which is then partially decomposed in the bladder to the form of bacteriologically active free colistin [3]. For the therapy of lower urinary tract infections (i.e., bladder infections, cystitis), CMS might have some value, even though most cystitis patients receive oral antibiotics and are successfully treated in the primary care. However, the pharmacokinetics of CMS, including its hydrolysis to active drug, shows substantial interpatient variability and this makes the choice of the proper dose difficult [3]. PMB has superior pharmacokinetic characteristics over CMS for the treatment of infections where rapid achievement and maintenance of the desired plasma concentration is important [3]. According to the results of this study, treatment with NAB739 is far superior since the compound can achieve both rapid and stable plasma concentration profile and high concentrations in urine.

Our findings are highly significant because over 6.3 million patients are yearly hospitalized in Europe, USA, and Japan due to cUTIs caused by *E. coli* and *K. pneumoniae* [14]. These two bacteria cause 80% of all cUTIs treated in the hospitals. It is estimated that in the future, many patients will likely be infected by extremely resistant strains. Therefore, novel polymyxins such as NAB739 and NAB815 might be very useful for the efficient treatment of cUTIs. The only potential drawback is that the use of polymyxins might be shadowed by already acquired resistance, even though this is still very rare [15]. Additional benefit in the case of NAB739 treatment is the characteristic for polymyxins ability to damage the outer membrane of polymyxin-resistant bacterial strains and, as a consequence, also the ability to sensitize bacteria to various “partner antibiotics” [16]. Whether combination of a polymyxin and a partner antibiotic will be effective in the case of cUTIs in the future, remains to be seen. 

## 5. Conclusions

The comparison of urinary levels of NAB739 and polymyxin B shows that even though subcutaneous administration of NAB739 and polymyxin B yield similar plasma concentrations in mice, only NAB739 is excreted in the urine at sufficient antibacterial concentration. The low efficacy of PMB against urinary tract infections demonstrated in previous studies is explained by the absence of compound in the murine urine even after the treatment at a high dose. These results explain higher efficacy of NAB739 than polymyxin B for the treatment of murine *E. coli* pyelonephritis. 

## Figures and Tables

**Table 1:** Multiple reaction monitoring (MRM) parameters for NAB739 and Polymyxin B.

Compound	MRM Transition	Cone Voltage, V	Collision Energy, eV
**NAB739**	359.66 > 425.19	30	8
Exact mass 1076 Da			
PMB *:	301.86 > 101.02	30	12
**Polymyxin B1**	402.12 > 101.02	30	15
Exact mass 1202 Da	602.50 > 101.02	30	30
	298.36 > 101.02	30	10
**Polymyxin B2**	301.44 > 101.02	30	15
Exact mass 1188 Da	595.42 > 101.02	30	30

* Polymyxin B (PMB) measured as a sum of polymyxin B1 and polymyxin B2.

**Table 2:** Polymyxin analysis method performance.

Validation Parameter	Performance Criteria	Obtained Value	
		NAB739	PMB
**Plasma**			
**Selectivity**	Response for the analyte in matrix blank ≤ 20% of LOQ	**2.3%**	**10%**
**Linearity**	R^2^ ≥ 0.985	R^2^ 0.9998	R^2^ 0.9998
		0.008–17.2 μg/mL	0.007–15.7 μg/mL
**Limit of quantification**	Set at concentration level where S/N ≥ 10	**0.008 μg/mL**	**0.007 μg/mL**
		(S/N ≥ 40)	(S/N ≥ 10))
**Urine**			
**Selectivity**	Response for analyte in matrix blank ≤ 20% of LOQ	**3.8%**	**10%**
**Linearity**	R^2^ ≥ 0.985	R^2^ 0.9996	R^2^ 0.998
		0.07–17.2 μg/mL	0.58–15.7 μg/mL
**Limit of quantification**	Set at concentration level where S/N ≥ 10	**0.07 μg/mL**	**0.58 μg/mL**
		(S/N ≥ 20)	(S/N ≥ 10))
